# *Campylobacter jejuni* Serine Protease HtrA Cleaves the Tight Junction Component Claudin-8

**DOI:** 10.3389/fcimb.2020.590186

**Published:** 2020-12-08

**Authors:** Irshad Sharafutdinov, Delara Soltan Esmaeili, Aileen Harrer, Nicole Tegtmeyer, Heinrich Sticht, Steffen Backert

**Affiliations:** ^1^ Department of Biology, Division of Microbiology, University of Erlangen-Nuremberg, Erlangen, Germany; ^2^ Division of Bioinformatics, Institute of Biochemistry, University of Erlangen-Nuremberg, Erlangen, Germany

**Keywords:** claudin-8, occludin, tight junction, E-cadherin, *Campylobacter*, protease, HtrA

## Abstract

*Campylobacter jejuni* express the high temperature requirement protein A (HtrA), a secreted serine protease, which is implicated in virulence properties of the pathogen. Previous studies have shown that *C. jejuni* HtrA can cleave the epithelial transmembrane proteins occludin and E-cadherin in the tight and adherens junctions, respectively. In the present report, we studied the interaction of HtrA with another human tight junction protein, claudin-8. Confocal immunofluorescence experiments have shown that *C. jejuni* infection of the intestinal polarized epithelial cells *in vitro* leads to a relocation of claudin-8. Wild-type *C. jejuni* induced the downregulation of claudin-8 signals in the tight junctions and an accumulation of claudin-8 agglomerates in the cytoplasm, which were not seen during infection with isogenic Δ*htrA* knockout deletion or protease-inactive S197A point mutants. Western blotting of protein samples from infected *vs*. uninfected cells revealed that an 18-kDa carboxy-terminal fragment is cleaved-off from the 26-kDa full-length claudin-8 protein, but not during infection with the isogenic Δ*htrA* mutant. These results were confirmed by *in vitro* cleavage assays using the purified recombinant *C. jejuni* HtrA and human claudin-8 proteins. Recombinant HtrA cleaved purified claudin-8 *in vitro* giving rise to the same 18-kDa sized carboxy-terminal cleavage product. Mapping studies revealed that HtrA cleavage occurs in the first extracellular loop of claudin-8. Three-dimensional modeling of the claudin-8 structure identified an exposed HtrA cleavage site between the amino acids alanine 58 and asparagine 59, which is in well agreement with the mapping studies. Taken together, HtrA operates as a secreted virulence factor targeting multiple proteins both in the tight and adherens junctions. This strategy may help the bacteria to open the cell-to-cell junctions, and to transmigrate across the intestinal epithelium by a paracellular mechanism and establish an acute infection.

## Introduction

*Campylobacter jejuni* is a Gram-negative, microaerophilic, spiral-shaped, flagellated proteobacterium that commensally colonizes the mucus layer in the lower intestinal tract of many birds and various mammals (Burnham and Hendrixson, 2018). The genus *Campylobacter* is evolutionarily related to the gastric pathogen *Helicobacter pylori* and considered to be the most common bacterial cause of human gastroenteritis in the world (Marder et al., 2017). *C. jejuni* represents a major zoonotic pathogen, which can be transmitted *via* a fecal > oral route through the consumption of contaminated poultry meats as well as other animal-derived food products (Hale et al., 2012). The rates of gastroenteric infections caused by *C. jejuni* are very high both in developed and developing countries (Kaakoush et al., 2015). Upon infection, *C. jejuni* appears to present lipooligosaccharides (LOS) at the bacterial cell surface, which in some patients can cross-react with human gangliosides resulting in autoimmune disorders such as the Guillian-Barré or Miller-Fisher syndromes (Ang et al., 2002; Yuki et al., 2004; Charles et al., 2017). Furthermore, in some cases this pathogen can promote colorectal cancer tumorigenesis through the action of microbial cytolethal distending toxin (CDT) (Brauner et al., 2010; He et al., 2019). Interestingly, CDT from *C. jejuni* was also extensively studied as an anti-cancer therapeutic agent for potential clinical applications (Lai et al., 2016). The pathogenic process generated by *C. jejuni* in the human intestine develops upon reaching the gut, where the bacteria attach to and then invade into epithelial cells, resulting in host tissue damage (Van Spreeuwel et al., 1985; Wooldridge and Ketley, 1997). Various bacterial adhesion proteins (adhesins) provide stable attachment through specific interaction with host cell receptors, which is a necessary requirement for subsequent host cell entry (Hermans et al., 2011; Backert et al., 2013). Several major adhesins were identified and have been reported to provide effective *C. jejuni* adhesion to a host cell, with the CadF (*Campylobacter* adhesin to fibronectin) protein being central in this process (Konkel et al., 1997; Schmidt et al., 2019). However, other adhesins also play important roles in cell attachment and include FlpA (fibronectin like protein A), JlpA (*jejuni* lipoprotein A), PEB1 (periplasmic binding protein 1), MOMP (major outer membrane protein), and some others (O Cróinín and Backert, 2012). Upon attachment, *C. jejuni* can enter host target cells *via* a signaling process involving the small Rho GTPases Rac1 and Cdc42 (Krause-Gruszczynska et al., 2007a; Krause-Gruszczynska et al., 2007b; Boehm et al., 2011; Krause-Gruszczynska et al., 2011; Eucker and Konkel, 2012). In addition, *C. jejuni* utilizes its flagellum as a type III secretion system (fT3SS) for the secretion or injection of effector proteins that interfere with host cell functions (Young et al., 1999; Christensen et al., 2009; Barrero-Tobon and Hendrixson, 2012). Furthermore, *C. jejuni* can transmigrate to the basolateral site of the intestinal epithelium by disrupting the host cellular junctions, and serine protease HtrA appears to play a driving role in this process (Boehm et al., 2018; Harrer et al., 2019).

HtrA proteins represent ATP-independent serine type proteases and are widely distributed both in prokaryotic and eukaryotic organisms (Clausen et al., 2002; Neddermann and Backert, 2019). Many bacteria can encode either one or more HtrA homologs (Li et al., 1996; Humphreys et al., 1999; Cortes et al., 2002; Purdy et al., 2002; Mo et al., 2006; Wilson et al., 2006; Ye et al., 2016). HtrA proteins due to their structure combine both protease and chaperone functions (Clausen et al., 2011). Bacterial HtrAs consist of an amino-terminal signal peptide, a trypsin-like serine protease domain and one or two PDZ-domains at the carboxy-terminus that regulate interactions with itself or other proteins (Kim and Kim, 2005; Skorko-Glonek et al., 2013). *Escherichia coli* is considered to be the best studied model organism concerning HtrA. This species encodes as many as three HtrA homologs, namely, DegP, DegQ, and DegS (Kim and Kim, 2005; Clausen et al., 2011). Their main function is to protect the bacterium against heat and other stresses, as well as to remove misfolded proteins. For example, inactivation of the *htrA* gene in *Streptococcus mutans* has been shown to affect its resistance to low and high temperatures, low pH as well as oxidative and DNA damaging agents (Biswas and Biswas, 2005). HtrAs can also play a major role in the pathogenesis of other Gram-positive and Gram-negative microbes (Backert et al., 2018). For example, the human pathogen *Streptococcus pyogenes* with impaired HtrA function expressed reduced amounts of mature streptococcal pyrogenic exotoxin B (SpeB), as it was shown by Western blot analysis and protease assays (Cole et al., 2007). In addition, *H. pylori* was the first bacterium shown to secrete HtrA into the extracellular environment, which was associated with the paracellular transmigration of the pathogen through cleavage of the host adherens junction protein E-cadherin (Harrer et al., 2018).

*Campylobacter jejuni* encodes one HtrA homolog, whose function was analysed by biochemical assays *in vitro* and in the bacteria *in vivo* (Brondsted et al., 2005; Zarzecka et al., 2020). Cryo-electron microscopy revealed the architecture of *C. jejuni* HtrA defined as a dodecamer, assembled by four trimers (Zarzecka et al., 2020). However, HtrA of *C. jejuni* can be also secreted into the extracellular space, where it has been shown to cleave the extracellular domain of E-cadherin at various positions (Boehm et al., 2012; Hoy et al., 2012). As a result, this helps *C. jejuni* to transmigrate between neighbouring host cells to the basal side of polarized gut epithelium. However, prior to the reach adherence junctions, *C. jejuni* faces the tight junction barrier, consisting of several proteins including tricellulin, occludin, claudins, and junction adhesion molecules (JAMs) (Guttman and Finlay, 2009; Gunzel and Yu, 2013; Van Itallie and Anderson, 2014; Slifer and Blikslager, 2020). This multiprotein junctional complex maintains “fence” tasks providing cell polarity and “gate” function, which provides selective transport of small molecules through the apical-basal barrier (Zihni et al., 2016). In addition, tight junction transmembrane proteins bind to intracellular scaffold proteins such as zonula occludens (ZO) -1, -2, and -3 forming tight connection with the actin cytoskeleton (Zihni et al., 2016). More recently, we have shown that *C. jejuni* can disrupt the tight junction protein occludin in an HtrA-dependent manner (Harrer et al., 2019). However, it is still unclear, whether other tight junction proteins such as the claudins may also be affected during *C. jejuni* paracellular transmigration.

Claudins represent tight JAMs responsible for the paracellular barrier function and account for at least 27 members in mammals (Tsukita et al., 2019). Based on their sequence homology, claudin family members consist of four putative transmembrane segments, a large extracellular loop (ECL1) containing a consensus sequence motif, and a second shorter extracellular loop (ECL2) also known as extracellular segments 1 and 2, respectively (Gunzel and Yu, 2013). Interestingly, *C. jejuni* has been shown to disrupt tight junctions during bacterial invasion of non-tumorigenic canine intestinal epithelial cells through claudin-4 cleavage (Lamb-Rosteski et al., 2008). Fluorescence microscopy revealed that infection of the epithelial monolayer with *C. jejuni* results in disruption of pericellular claudin-4, while Western blotting showed significantly less total claudin-4 (Lamb-Rosteski et al., 2008). However, the molecular background of claudin degradation during *C. jejuni* infection remained unclear. *C. jejuni* has been already shown to disrupt the major junction proteins occludin and E-cadherin during paracellular migration in an HtrA-dependent manner (Boehm et al., 2012; Harrer et al., 2019), which may be associated with shedded outer-membrane vesicles (Elmi et al., 2018). The results presented here suggest that claudin-8 is a major novel cleavage target for *C. jejuni* HtrA, and besides occludin, the second target protein in the tight junctions, which may help the pathogen to disrupt the epithelial barrier during infection.

## Materials and Methods

### *Campylobacter* Strains and Infection Assays

The *C. jejuni* wild-type (wt) strain 81-176 and its isogenic knockout mutant *C. jejuni ΔhtrA*, the complemented mutant *ΔhtrA/htrA* and protease-inactive S197A point mutation in the *htrA* gene were used throughout this study (Boehm et al., 2012; Boehm et al., 2015). Bacterial cells were cultured using *Campylobacter* blood-free selective agar base including *Campylobacter* growth supplement provided by Oxoid (Wesel, Germany). Alternatively, the bacteria were grown on Mueller-Hinton (MH) agar supplemented with chloramphenicol (20 μg/ml) or kanamycin (30 μg/ml), respectively. Incubation was for 48 h at 37°C in jars using microaerobic conditions provided by the CampyGen™ system from Oxoid. All *C. jejuni* strains were harvested using sterile cotton swabs and resuspended in liquid BHI medium. The optical density (OD) was measured at 600 nm in an Eppendorf spectrophotometer to calculate the number of bacterial cells followed by host cell infection of *C. jejuni* using a multiplicity of infection (MOI) of 100.

### Cell Culture and Immunofluorescence Staining

The human intestinal cell lines Caco-2 (ATCC HTB-37) and T84 (ATCC CCL-248) were seeded into 75 cm^2^ cell culture flasks and finally in 12-well plates using DMEM medium including 10% FCS (Invitrogen) and 4 mM glutamine (Invitrogen, Karlsruhe, Germany). The cells formed proper monolayers and were incubated for 14 days to allow proper cell polarization. After a 12-h co-incubation with *C. jejuni*, the infected cells were washed twice with sterile PBS buffer followed immunofluorescence staining according to a previous protocol (Krause-Gruszczynska et al., 2007a). In brief, the cells have been fixed for 10 min in 4% PFA (paraformaldehyde) at 20°C. Afterwards, the cells were permeabilized for 1 min using 0.25% Triton-X100 and then blocked for 1 h in PBS buffer containing 5% BSA. Immunostaining of the cells was performed with the following antibodies: α-claudin-8 (#710222 and #40-0700Z, Invitrogen) and α-occludin (#sc-133256, Santa Cruz Biotechnology). The *C. jejuni* bacteria were visualized by α-*Campylobacter* antibody (Dako, Glostrup, Denmark). The DNA in the nucleus was stained by DAPI (4’-6-diamidino-2-phenylindole dihydrochloride) (Thermo Fisher Scientific). TRITC (tetramethylrhodamine isothiocynate)-conjugated α-rabbit, TRITC-conjugated α-mouse, FITC (fluorescein isothiocyanate)-conjugated α-rabbit and Alexa-633-conjugated α-rabbit (Thermo Fisher Scientific) were utilized as secondary antibodies. All samples were investigated by confocal fluorescence microscopy using a Leica SP5 (Leica Microsystems, Wetzlar, Germany). Excitation/emission of the fluorescence from DAPI, FITC and TRITC was processed at 405/413–460 nm, 488/496–550 nm, and 561/571–630 nm wavelengths, respectively. The obtained data were visualized using LAS AF computer software (Leica Microsystems). All microscopic experiments were performed at the Optical Imaging Centre Erlangen (OICE, Erlangen, Germany).

### Quantification of the Fluorescence Intensities of Claudin-8 and Occludin

The images of T84 cells after confocal immunofluoresence microscopy were subjected for further analysis in the Fiji platform (Schindelin et al., 2012). Cellular localization of claudin-8 and occludin in the T84 cell monolayers was analyzed by segmentation of the tight junctions area and the cytoplasm area into regions of interest (ROIs). In the defined ROIs (tight junctions or cytoplasm), we quantified the relative fluorescence units (RFUs) of claudin-8 and occludin, respectively. The mean value of RFUs for each condition (claudin-8 in tight junctions vs. claudin-8 in the cytoplasm, occludin in tight junctions vs. occludin in the cytoplasm) was calculated from 10 cells in the T84 monolayer and presented as mean ± standard deviation. In addition, the fluorescence intensity of cells was assessed in single representative T84 cells (mock control cells or cells infected with wt *C. jejuni*). The RFUs were counted within a straight line passing through a cell including tight junctions and the cytoplasm areas, as marked, between the yellow arrows of **Figure 3B**.

### Cloning, Expression, and Purification of *C. jejuni* HtrA

Recombinant *C. jejuni* HtrA was purified under native conditions as described previously (Zarzecka et al., 2018). For this purpose, *C. jejuni* HtrA of strain 81-176 without the signal peptide (amino acids 17-472) was amplified from genomic DNA and cloned in the expression plasmid pGEX-6P-1 (GE Healthcare Life Sciences, Munich, Germany) as a GST-fusion protein using the restriction sites *Bam*HI and *Xma*I. The expression was performed in *Escherichia coli* BL21 and the purification protocol was described previously in detail (Löwer et al., 2008). *E. coli* LPS has been removed by incubating 100 μg/ml of HtrA for 1 h using 10 μg/ml of polymyxin B (Sigma Aldrich) at 20°C (Brisslert et al., 2005). The final purity of HtrA was determined to be more than 95% by SDS-PAGE electrophoresis and Coomassie staining (Moese et al., 2001).

### *In Vitro* HtrA Cleavage Assays

Cleavage assays were performed with recombinant *C. jejuni* HtrA and purified human full-length Claudin-8 coupled to GST (# H00009073-P01, Abnova, Taipei City, Taiwan). For this purpose, 100 ng of GST-claudin-8 were incubated with 30 ng of purified *C. jejuni* HtrA in 25 µl 50 mM HEPES buffer (pH 7.4) for 16 h at 37°C. The resulting cleavage products were analysed by SDS-PAGE and Western blotting using α-claudin-8, α-GST, and α-HtrA antibodies as described below.

### Western Blotting Studies

Proteins derived from infected cells and *in vitro* HtrA cleavage assays were loaded on 8% SDS-PAGE gels and blotted on PVDF membranes as described (Hartung et al., 2015). Afterwards, the membranes were prepared for blocking for 1 h using either 3% BSA or 5% skim milk in TBST (25 mM Tris-HCl pH 7.4, 140 mM NaCl, 0.1% Tween-20) buffer at 20°C. The following antibodies were used: rabbit α-claudin-8 (#710222, Invitrogen), mouse α-GAPDH (Sigma Aldrich, Taufkrichen, Germany), or rabbit α-HtrA (Brondsted et al., 2005). These antibodies were incubated overnight at 4°C using the manufacturer’s or published protocols. Horseradish peroxidase-conjugated α-rabbit or polyvalent α-mouse immunoglobulin were utilized as secondary antibodies (Life Technologies, Darmstadt, Germany). The detection of bound antibodies was accomplished using the ECL Plus chemiluminescence Western Blot kit (GE Healthcare) (Hirsch et al., 2012).

### Bioinformatics

The sequence logo describing the HtrA cleavage site was generated using the “frequency plot” option of WebLogo (Crooks et al., 2004). The search for HtrA cleavage sites in claudin-8 was done with ScanProsite (de Castro et al., 2006). Modeling of the claudin-8 structure was performed with HHpred (Zimmermann et al., 2018) and Modeller (Webb and Sali, 2017) using the structure of Claudin-9 (Vecchio and Stroud, 2019) as a template. RasMol (Sayle and Milnerwhite, 1995) was used for structure analysis and visualization.

### Statistics

All data were evaluated *via* two-tailed Mann-Whitney test with GraphPad Prism 6 (Version 6.01). The obtained p-values *p < 0.001* (***) and *p < 0.0001* (****) were defined as statistically significant; ns, non-significant.

## Results

### Rearrangement of Claudin-8 in the Tight Junctions of Polarized Intestinal Epithelial Cell Lines by *C. jejuni* HtrA

**Figure 1:**
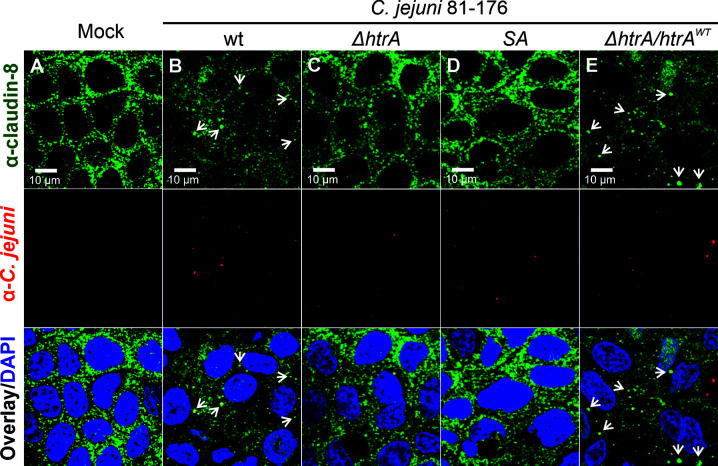
Infection of polarized Caco-2 epithelial cell monolayers by *C. jejuni* 81-176 results in the disruption of claudin-8 patterns. Epithelial cells were immunostained with α**-**claudin-8 (green) and α-*C. jejuni* (red) without infection **(A)**, or after infection with *C. jejuni* wild-type (wt) strain **(B)**, Δ*htrA* knockout mutant **(C)**, Δ*htrA* complemented with protease inactive SA mutant **(D)** or wt *htrA* **(E)**. Infection was performed for 12 h at an MOI of 100. DAPI staining (blue) was used for the nuclear DNA counterstaining. White arrows indicate claudin-8 redistribution to agglomerates.

To evaluate whether *C. jejuni* HtrA affects the claudin-8 distribution in the cellular tight junctions, immunofluorescence microscopy has been applied. For this purpose, confluent Caco-2 epithelial cells were grown in monolayers for 14 days to achieve proper polarization and detectable expression of claudin-8. The cells were then infected either with *C. jejuni* wt strain 81-176 or its isogenic Δ*htrA* knockout mutant. As control experiments, Caco-2 cells were infected with *C. jejuni* 81-176 Δ*htrA* complemented either with wt *htrA* (Δ*htrA*/*htrA* ^wt^) or a protease-inactive S197A point mutant (Δ*htrA*/*htrA* ^SA^). After 12 h of infection, Caco-2 cells were fixed in PFA and immunostained with antibodies against claudin-8 and *C. jejuni* as labelled with green and red fluorophores, respectively. Immunofluorescence microscopy revealed that non-infected Caco-2 monolayers are characterized by the uniform distribution of claudin-8 in the tight junction areas (**Figure 1A**), while infection with wt *C. jejuni* or the wt complemented strain (Δ*htrA*/*htrA* ^wt^) led to mislocalization of claudin-8 out of the tight junctions to form agglomerates in the cytoplasm (**Figures 1B, E**, white arrows). Interestingly, when Caco-2 monolayers were infected with *C. jejuni* carrying deleted *htrA* or the protease-inactive *htrA* ^S197A^ point-mutated gene, claudin-8 localization within cellular tight junctions was not or only slightly affected (**Figures 1C, D** **)**.

**Figure 2:**
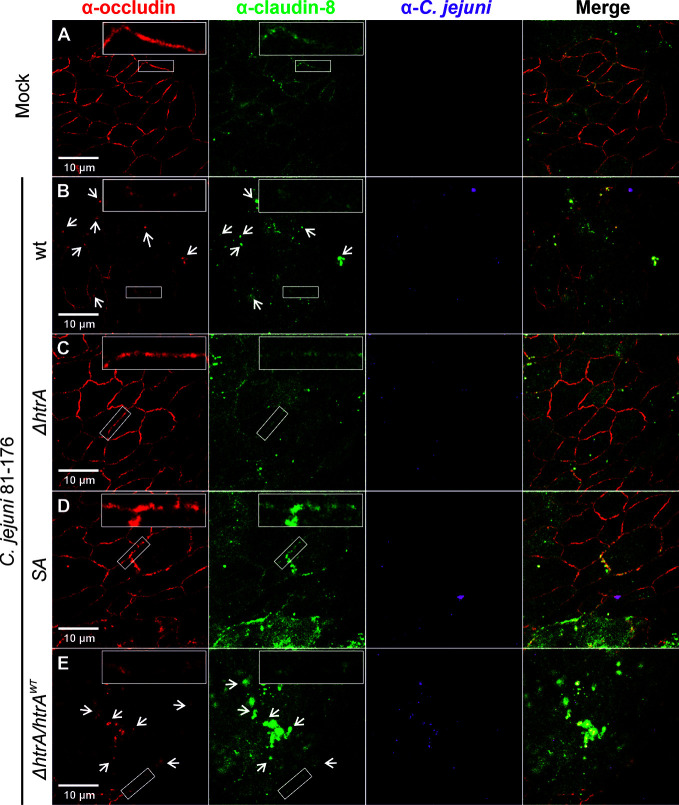
Infection of polarized T84 epithelial cell monolayers by *C. jejuni* 81-176 leads to disruption of claudin-8 and occludin in the tight junctions. Epithelial cells were immunostained with α-occludin (red), α-claudin-8 (green) and α-*C. jejuni* (magenta) without infection **(A)**, or after infection with *C. jejuni* wild-type (wt) strain **(B)**, Δ*htrA* knockout mutant **(C)**, protease inactive SA mutant **(D)** or Δ*htrA* complemented with wt *htrA* **(E)**. White arrows indicate occludin and claudin-8 redistribution to protein agglomerates. White boxes contain enlarged parts of tight junctions from corresponding smaller white boxes.

To corroborate the above findings with claudin-8, we utilized a second intestinal epithelial cell line, T84. To this end, T84 cell monolayers were grown for 14 days and then infected with the above described *C. jejuni* strains under identical conditions. Besides claudin-8, we additionally counterstained the cells with the tight junction protein occludin, which we have shown recently to be cleaved by *C. jejuni* HtrA (Harrer et al., 2019). As expected, in non-infected T84 control cells or cells infected with *C. jejuni* carrying a defective *htrA* gene, both proteins (occludin and claudin-8) revealed similar staining patterns in the cellular tight junctions (**Figures 2A, C, D** **)**. In contrast, infection with wt *C. jejuni* or the wt complemented strain (Δ*htrA*/*htrA* ^wt^) led to the disruption of both occludin and claudin-8 in tight junctions along with their appearance in the cytoplasmic area (**Figures 2B, E**, white arrows). The inlays on top of each panel show enlarged sections of tight junctions from corresponding areas marked with smaller white boxes.

### Quantification of Claudin-8 Signals in the Tight Junctions and Cytoplasm During *C. jejuni* Infection

**Figure 3:**
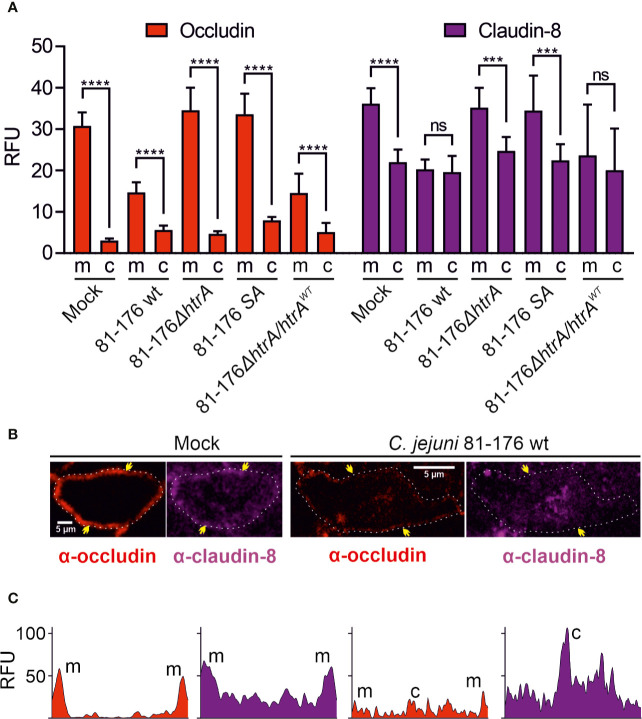
Localization of occludin and claudin-8 proteins in tight junctions and cytoplasm of T84 cells. The mean florescence intensity of the proteins was assessed separately in the membrane (m)-associated tight junctions and cytoplasm (c), and was calculated from 10 T84 cells per sample **(A)**. Single cell analysis of representative T84 cells without or with infection by wt *C. jejuni*. Infection of T84 cells leads to the drop of fluorescence intensity of claudin-8 in tight junctions along with its appearance in the cytoplasm **(B)**. Corresponding fluorescence intensity plots for occludin and claudin-8 within single cells between two yellow arrows, as marked in panel **(B)**. When infected with wt *C. jejuni*, a strong fluorescence intensity can be found in the cell cytoplasm **(C)**. RFU, relative fluorescence units of membrane and cytoplasmic localization. ***p < 0.001, ****p < 0.0001, ns, non-significant.

To further analyze the distribution of occludin and claudin-8 in the host cells, we quantified the mean fluorescence intensity of the proteins in the areas of membrane-associated tight junctions and cytoplasm within the overall cell population. In mock control cells, both occludin and claudin-8 revealed a higher fluorescence intensity within the tight junctions compared to the cytoplasmic proteins (**Figure 3A**). Similar patterns of occludin and claudin-8 in the tight junctions were found in T84 cells infected with *C. jejuni* carrying an impaired *htrA* gene. In contrast, infection of T84 cells with wt *C. jejuni* or the wt complemented strain led to a significant drop of fluorescence intensity of occludin and claudin-8 in the cellular tight junctions. In particular, the mean fluorescence of cytosolic occludin slightly increased when infected with wt *C. jejuni* (*p < 0.0001*) or with the wt complemented strain (*p < 0.01*), which is in agreement with our previous studies (Harrer et al., 2019). The mean fluorescence of cytosolic claudin-8 upon infection did not change significantly within overall cell populations. However, the bright fluorescent aggregates of the cytosolic claudin-8 were only detected in T84 cells infected with wt *C. jejuni* carrying an intact *htrA* gene, confirming the re-distribution of the protein from the cell membrane (**Figure 2**, white arrows and **Figure 3B**). The fluorescence intensity of cells was further assessed in representative single cells between the yellow arrows as marked in **Figure 3B**. In non-infected T84 cells the peaks of fluorescence for occludin and claudin-8 appeared in the cell periphery, suggesting their prevailing membrane localization (**Figure 3C**). After infection of T84 cells with wt *C. jejuni*, the fluorescence intensity of occludin and claudin-8 at the cell periphery was significantly reduced. Moreover, in infected T84 cells the strong fluorescence signals of claudin-8 (comparable to the fluorescence of the protein in the membrane of non-infected cells) were also detectable in the cytoplasm (**Figure 2**, white arrows, and **Figure 3C**). Based on these immunofluorescence experiments, we can conclude that HtrA is involved in the disturbance of claudin-8 and its translocation from tight junctions into the cytoplasm during infection with *C. jejuni*.

### HtrA Induces Claudin-8 Cleavage During *C. jejuni* Infection of Polarized Intestinal Epithelial Cells

**Figure 4:**
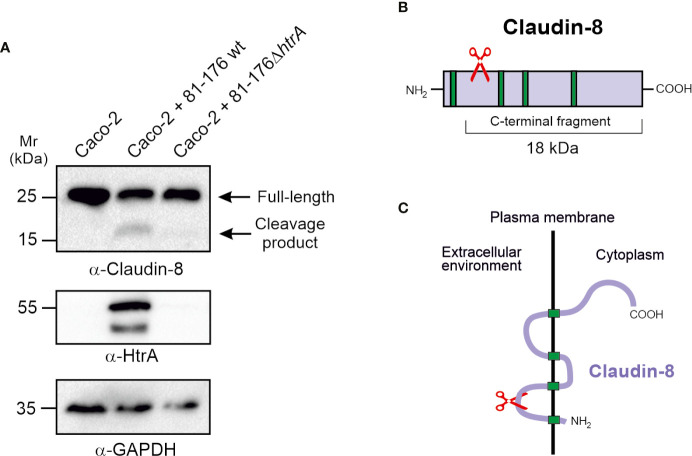
HtrA cleaves claudin-8 during *C. jejuni* infection *in vivo*. **(A)** Immunoblotting of protein extracts from polarized Caco-2 cell monolayers infected with *C. jejuni* wild-type (wt) or Δ*htrA* mutant. The blots were stained with polyclonal α-claudin-8 antibodies recognizing the carboxy-terminus of the protein. The α-HtrA and α-GAPDH blots served as controls. Besides the full-length protein (~26 kDa), a cleaved carboxy-terminal fragment (~18 kDa) was visualized with α- claudin-8 antibodies upon infection with *C. jejuni* wt. **(B)** Proposed cleavage site position in the amino-terminus of claudin-8 for HtrA protease according to the size of protein cleavage products determined by SDS-PAGE marker proteins. **(C)** A model for claudin-8 cleavage in the first extracellular loop (ECL1) by HtrA protease.

Since infection by *C. jejuni* expressing intact HtrA leads to disorganisation of claudin-8 in Caco-2 cells, we came into assumption that HtrA might have a specific proteolytic activity against claudin-8. To test whether *C. jejuni* HtrA is inherently involved in claudin-8 proteolytic cleavage, immunoblotting assays were used. Polarized Caco-2 monolayers were infected with either wt *C. jejuni*, expressing intact HtrA or Δ*htrA* deletion mutant. After 12-h infection, immunoblotting was applied by using α-claudin-8, α-HtrA and α-GAPDH antibodies (**Figure 4A**). When infected with wt *C. jejuni* we observed full-length claudin-8 at about 26 kDa, and an additional band of lower intensity that appeared at approximately 18 kDa. In contrast, Caco-2 cells that were exposed to *htrA*-deficient *C. jejuni*, an 18-kDa band was not detected, assuming the absence of cleavage. The immunogenicity of α-claudin-8 antibody is directed against amino acids 206–225 at the carboxy-terminus of the protein, which corresponds to the QKSYHTGKKSPSVYSRSQYV sequence. Since the antibody recognized an 18-kDa claudin-8 fragment, we proposed that this fragment corresponds to the carboxy-terminus and cleavage of claudin-8 by *C. jejuni* HtrA takes place at the amino-terminus of the protein, presumably located in the first extracellular loop (**Figures 4B, C** **)**. We were not able to detect the remaining very small amino-terminal cleaved peptide of claudin-8 due to lack of antibodies against this part of the amino-terminal tail.

### *C. jejuni* HtrA Cleaves Claudin-8 by an *In Vitro* Cleavage Assay

**Figure 5:**
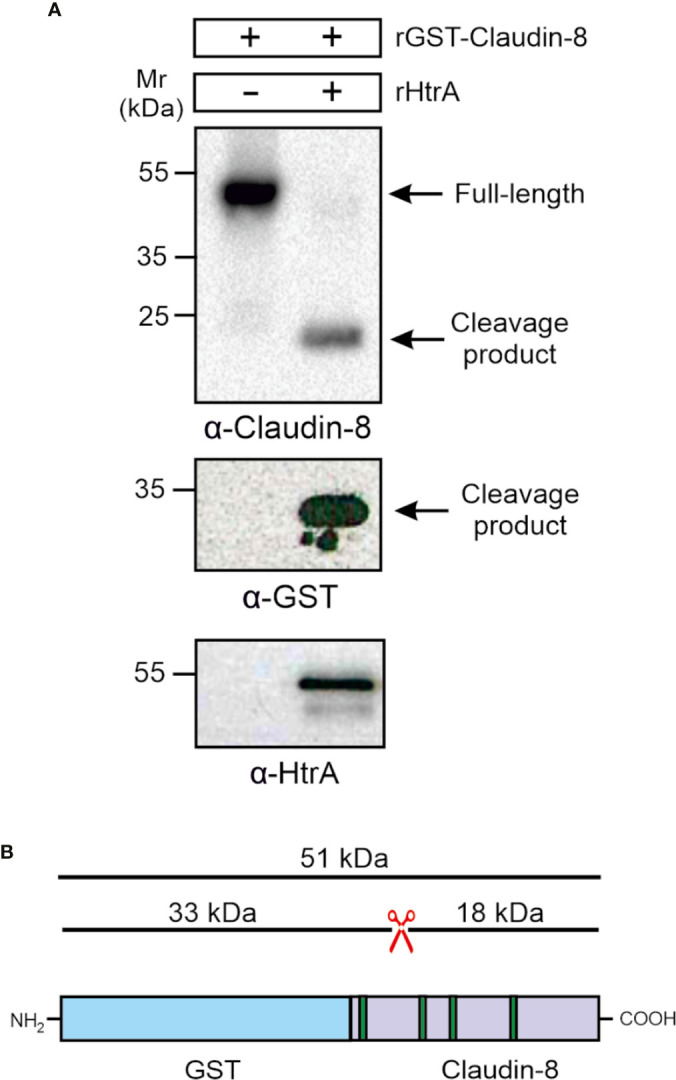
HtrA cleaves claudin-8 in a cleavage assay *in vitro*. **(A)** Recombinant GST-tagged human claudin-8 was incubated with either purified rHtrA *in vitro*. Immunoblotting against α-claudin-8 showed cleavage of the full-length GST-tagged claudin-8 protein, when incubated with recombinant HtrA resulted in the appearance of an 18-kDa claudin-8 fragment. In addition, a cleaved 33-kDa fragment was visualized in the blot with α-GST antibodies. **(B)** Mapping of the GST-claudin-8 cleavage fragments. The full-length GST-tagged claudin-8 protein (51 kDa) is cleaved into a carboxy-terminal 18-kDa fragment of claudin-8 and a 33 kDa amino-terminal fragment containing the GST-tag and the remaining 8-kDa amino-terminal fragment of claudin-8.

To confirm whether the carboxy-terminal 18-kDa fragment of claudin-8 cleavage is the result of direct HtrA proteolytic activity, and not by any other bacterial or cellular protease, an *in vitro* cleavage assay using the recombinant proteins was conducted. For this purpose, we utilized recombinant amino-terminally GST-tagged claudin-8 (rGST-Claudin-8, about 51 kDa), which was incubated with purified *C. jejuni* HtrA for 12 h at 37°C. The *in vitro* cleavage reactions were subjected to immunoblotting using α-claudin-8, α-GST and α-HtrA antibodies. The immunostaining with α-claudin-8 revealed the appearance of the same sized carboxy-terminal 18-kDa fragment of claudin-8 through cleavage by HtrA (**Figure 5A**). GST is a protein of 25 kDa. The remaining N-terminal fragment was detected after staining with α-GST at 33 kDa, which corresponds to the GST protein fused to the cleaved 8-kDa amino-terminus of claudin-8 (**Figures 5A, B** **)**.

### Mapping of the *C. jejuni* HtrA Cleavage Site for Claudin-8 Proteolysis

**Figure 6:**
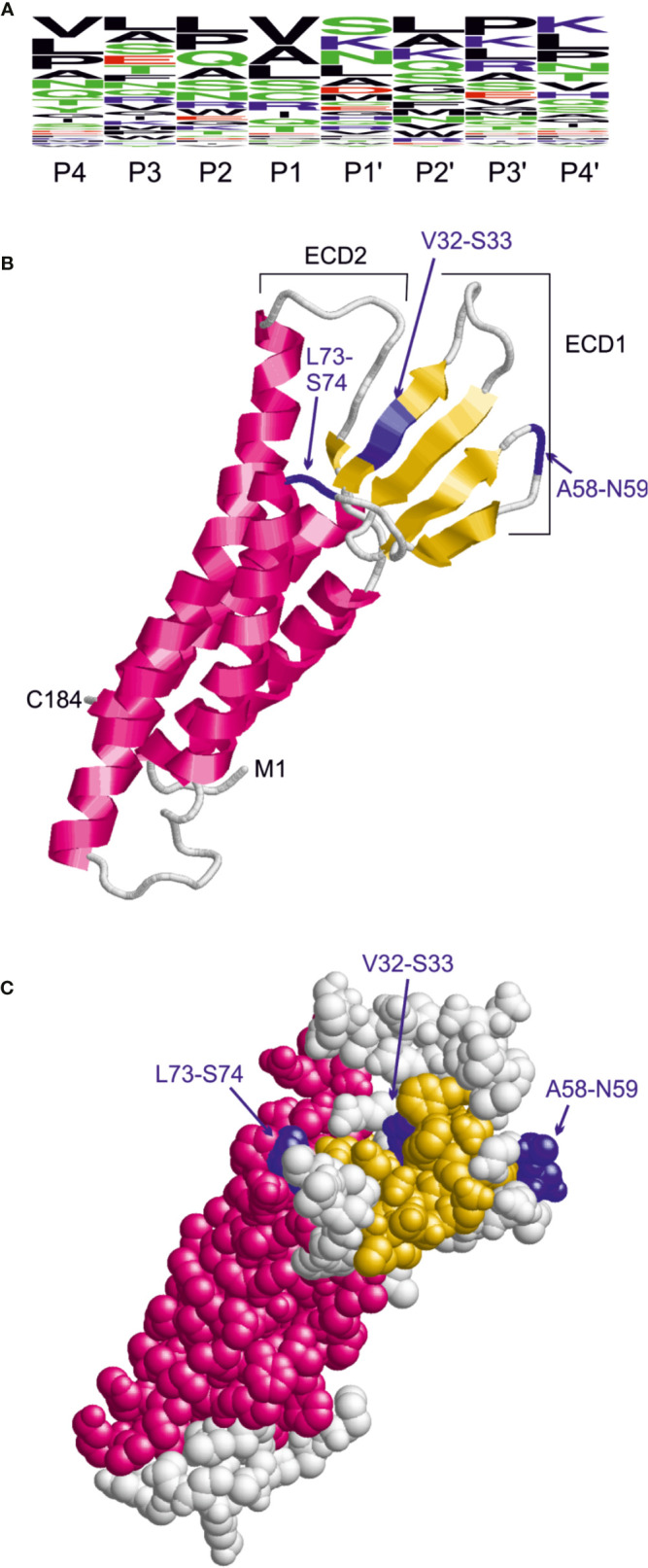
Identification of candidate HtrA cleavage sites in human claudin-8. **(A)** Sequence logo derived from the HtrA cleavage sites in β-casein and lysozyme. The height of the letters is proportional to the relative frequency of the amino acid at the respective sequence position (P4 to P4’) denote the individual positions of the HtrA cleavage site. **(B)** Model of the claudin-8 structure indicating the position of candidate cleavage sites (blue) in the extracellular domain 1 that confer the pattern [VAL]-[SKN]. **(C)** The space-filled-presentation of claudin-8 shows that only the A58-N59 site is fully accessible for recognition by a protease.

The sequence preferences for *C. jejuni* HtrA cleavage were identified recently based on the cleavage sites detected in the β-casein and lysozyme substrates by mass spectrometry (Zarzecka et al., 2020). This analysis revealed that the cleavage site exhibits a rather large sequence heterogeneity and that the highest conservation is observed for the P1 and P1’ sites. Based on the three predominant residues observed at the P1 and P1’ site, we defined the pattern [VAL]-[SKN] to search for HtrA cleavage sites in the extracellular domain 1 (ECD1) of claudin-8. A frequency plot of the HtrA cleavage sites was established (**Figure 6A**). The height of each character is proportional to the frequency of the amino acid residue at the individual position of the cleaved peptide. This analysis revealed that there are three instances of this pattern in ECD1, namely, V32-S33, A58-N59, and L73-S74. The location of these sites in the claudin-8 structure is shown in **Figures 6B, C**. The V32-S33 site is buried in a β-sheet and therefore only hardly accessible to HtrA cleavage. The L73-S74 site is located in the immediate vicinity to a transmembrane helix; therefore, cleavage is sterically hindered by the presence of the membrane. In contrast, A58-N59 is located in an exposed turn connecting two β-strands and therefore is accessible. Thus, the A58-N59 position represents the most likely site for *C. jejuni* HtrA cleavage in claudin-8.

## Discussion

The tight junctions in the intestine play a major role both in epithelial cell monolayer integrity and in selective transport of molecules across neighboring epithelial cells. The key proteins building the tight junction net include claudin family members, occludin and tricellulin that localize at the upper tricellular contacts facing the lumen. During infection, some microbial pathogens can target the tight junction complex leading to protein dysregulation, which results in the disruption of intestinal barrier homeostasis in order to support microbial survival, spread and sometimes persistence (Vogelmann et al., 2004). For example, the human pathogen *Vibrio cholerae* secretes a metalloprotease, haemagglutinin/protease (HA/P), which degrades the occludin extracellular domain and subsequently affects the host cell actin cytoskeleton due to impaired interactions with the scaffold protein ZO-1 (Schubert-Unkmeir et al., 2010). *Neisseria meningitidis* was shown to activate matrix metalloproteinase 8 in human brain microvascular endothelial cells, resulting in cleavage of occludin. This resulted in disappearance of occludin from the cell periphery and cleavage to a lower-sized 50-kDa protein (Schubert-Unkmeir et al., 2010). Finally, multiple Gram-negative bacteria including *C. jejuni*, *H. pylori*, *Escherichia coli*, and *Shigella flexneri* secrete the HtrA protease towards adherence junctions, where it cleaves the adherens junction receptor protein E-cadherin (Schmidt et al., 2016). Recently, *C. jejuni* HtrA was shown to cleave E-cadherin and occludin upon infection of intestinal polarized Caco-2 cells (Harrer et al., 2019). In our previous analysis, we have identified the cleavage sites of *H. pylori* HtrA in the E-cadherin protein with a consensus cleavage sequence occurring at the [VITA]-[VITA]-x-x-D-[DN] motif (Schmidt et al., 2016). We have then identified a simplified pattern [VITA]-[VITA]-x(2,4)-[DN] of an HtrA cleavage site in occludin, which occurred in the second extracellular loop and was characterized by lack of secondary structure (Harrer et al., 2019). The present results shown here demonstrate that HtrA from *C. jejuni* can cleave another major tight junction protein, claudin-8, as this has been shown both *in vitro* with purified proteins and upon infection of cultured polarized Caco-2 and T84 cells *in vivo*.

Claudins represent highly conserved 20- to 27-kDa proteins that are differentially expressed along the various epithelial compartments of the gastrointestinal tract (Kim et al., 2019). Claudins are composed of four transmembrane regions including a short intracellular amino-terminal sequence (~1–7 residues), a large first extracellular loop (~52 residues), a shorter second extracellular loop (16–33 residues), and a cytoplasmic carboxy-terminal domain that varies considerably in length between different isoforms (21–63 residues) (Kim et al., 2019). The function of these proteins is mainly determined by their extracellular loops ECL1 and ECL2 (Krause et al., 2008). When the larger ECL1 is supposed to provide paracellular tightness and the selective ion permeability, the smaller ECL2 might contribute in narrowing of the paracellular cleft and hold neighboring cell membranes (Wen et al., 2004; Piontek et al., 2008). The decreased barrier function due to disruption of tight junctions leads to alterations in levels of pro-inflammatory signaling that apparently results in variety of pathologies (Harhaj and Antonetti, 2004; Turner, 2006). Thus, dysregulation of claudins in the gastrointestinal tract can lead to various illnesses such as inflammatory bowel disease, celiac disease and gastroesophageal reflux disease (Zeissig et al., 2007; Szakal et al., 2010; Monkemuller et al., 2012). Claudin-8, in particular, has been shown to be expressed in the small and large intestines, liver and gallbladder and to be involved in the tight junction barrier function (Jeansonne et al., 2003; Kim et al., 2019). Downregulation and redistribution of claudin-8 along with claudin-5 lead to alterations in tight junction’s structure and pronounced barrier dysfunction both in mild and moderately active Crohn’s disease (Zeissig et al., 2007).

Since claudin-8 plays an important role in barrier function of intestinal epithelial cell monolayers, we aimed to elucidate if this tight junction protein could be a target for *C. jejuni* HtrA. We have previously shown that upon infection of Caco-2 wt cells, *C. jejuni* exploits the secreted serine protease HtrA to cleave the adherens junction protein E-cadherin (Boehm et al., 2012). Then, we have demonstrated that both the apical tight junction proteins occludin (Harrer et al., 2019) and claudin-8 (this work) are also disrupted by HtrA during *C. jejuni* infection facilitating pathogen entry into the intercellular space between neighboring cells of the gut epithelium. This approach may help the bacteria to transmigrate across the intestinal epithelium by a paracellular mechanism and reach basal surfaces and the fibronectin-integrin complex that connect epithelial cells with underlying tissue (Backert et al., 2013; Backert et al., 2018). In particular, *C. jejuni* uses the fibronectin and integrin receptors to enter host cells in a CadF/FlpA-dependent manner (Boehm et al., 2011; Krause-Gruszczynska et al., 2011; Eucker and Konkel, 2012). While some major junctional proteins such as claudin-8, occludin and E-cadherin are targeted by HtrA, *htrA*-deficient bacteria do not and are strongly diminished in transmigration, adhesion and invasion of polarized Caco-2 cells (Harrer et al., 2019).

While tight junctions are essential in regulating the permeability across the epithelia, tight junctions can also mediate signaling pathways in response to other factors, for instance, through phosphorylation (Nunbhakdi-Craig et al., 2002; Fujibe et al., 2004). This can directly regulate the permeability of the cell monolayer, by promoting the barrier function or increasing Mg^2+^ transport (Ishizaki et al., 2003; Ikari et al., 2008). For example, a mutant protein kinase WNK4 present in patients with the pseudohypoaldosteronism type II disorder was found to phosphorylate claudins 1-4 resulting in elevated paracellular permeability (Yamauchi et al., 2004). In general, phosphorylation of claudins appeared as a vital process required for the maintenance of cell homeostasis but this also makes it an attractive target for the pathogens. Though we did not determine the claudin-8 phosphorylation status in the present report, due to lack of corresponding antibodies, *C. jejuni* HtrA could either potentially affect phosphorylation-mediated downstream signaling *via* claudin-8 or directly cleave target proteins. This idea should be studied in more detail in future experiments. Finally, tight junction proteins such as the claudins can be targeted not only by *C. jejuni*, but also other microbial pathogens. For instance, *H. pylori* HtrA can also cleave claudin-8 resulting in the same 18-kDa carboxy-terminal fragment (Tegtmeyer et al., 2017). This suggests that *H. pylori* and *C. jejuni* HtrAs most likely cleave the same sequence between the A58-N59 position in claudin-8. In addition, *Clostridium perfringens* has been shown to destroy the epithelial cell layer through the interaction of an enterotoxin with the claudin-4 protein by using it as a host cell receptor (Eichner et al., 2017). In a similar way, *C. perfringens* enterotoxin has been found to interact with other claudin members including claudin-5, -6, -8 and -14, confirming that they share a similar structural topology (Lal-Nag et al., 2012; Shrestha and McClane, 2013; Liao et al., 2016). Thus, the claudins in the tight junctions represent preferred targets by multiple microbial pathogens.

Taken together, we found that *C. jejuni*, in addition to occludin and E-cadherin, is capable to cleave claudin-8, which results in the disruption of major junction proteins in an HtrA-dependent manner. Thereby, this microbial pathogen can reach the basal side of polarized epithelial cells by transmigration through the tight and adherens junctions disrupted by secreted HtrA. However, cleavage of occludin and claudin-8 by *C. jejuni* HtrA might be just one option for the paracellular transmigration, while another intriguing mechanism could be the control of potential phosphorylation of claudins triggered by bacterium. *C. jejuni* is widely known to hijack host molecular signaling for its own benefit, for instance, by phosphorylation of host cell receptors such as EGFR, PDGFR and other signaling proteins (Tegtmeyer et al., 2020), and further investigation of this strategy can provide new insights in the pathogenesis of these important bacteria.

## Data Availability Statement

The raw data supporting the conclusions of this article will be made available by the authors, without undue reservation.

## Author Contributions

IS, DS, and AH performed the infection studies. IS performed the immunofluorescence experiments. DS, AH, and NT did the *in vitro* cleavage experiments. HS performed the bioinformatics analysis and cleavage site identification. IS analyzed the data. SB conceptualized the study, analyzed the data, and wrote the paper together with IS. All authors contributed to the article and approved the submitted version.

## Funding

This work is supported by German Federal Ministries of Education and Research (BMBF) in frame of the zoonoses research consortium PAC-Campylobacter to SB (IP9/ 01KI1725E) and by the German Science Foundation (DFG) to NT (TE776/3-1). We thank the FAU University Erlangen for their support to publish Open Access papers. We thank the FAU University Erlangen for their support to publish Open Access papers.

## Conflict of Interest

The authors declare that the research was conducted in the absence of any commercial or financial relationships that could be construed as a potential conflict of interest.
